# Impact of Birth Weight and Length on Primary Hypertension in Children

**DOI:** 10.3390/ijerph16234649

**Published:** 2019-11-22

**Authors:** Aneta Weres, Joanna Baran, Ewelina Czenczek-Lewandowska, Justyna Leszczak, Artur Mazur

**Affiliations:** 1 Medical College, University of Rzeszów, 35-959 Rzeszów, Poland; joannabaran.ur@gmail.com (J.B.); e.czenczek@univ.rzeszow.pl (E.C.-L.); leszczakjustyna.ur@gmail.com (J.L.); drmazur@poczta.onet.pl (A.M.); 2 Clinical Regional Hospital No.2 in Rzeszów, Lwowska Street 60, 35-301 Rzeszów, Poland

**Keywords:** birth weight, birth length, blood pressure, hypertension

## Abstract

Background: A child’s birth parameters not only enable assessment of intrauterine growth but are also helpful in identifying children at risk of developmental defects or diseases occurring in adulthood. Studies show that children born with a body weight that is small for their gestational age (SGA) are at a greater risk of hypertension though the inverse relation between excessive birth weight and the risk of primary hypertension in children is discussed less frequently. Purpose: To assess the impact of both birth weight and length on hypertension occurring in children aged 3–15 years. Methods: A total of 1000 children attending randomly selected primary schools and kindergartens were examined. Ultimately, the analyses took into account n = 747 children aged 4–15; 52.6% boys and 47.4% girls. The children’s body height and weight were measured; their blood pressure was examined using the oscillometric method. Information on perinatal measurements was retrieved from the children’s personal health records. Results: Compared to the children with small for gestational age (SGA) birth weight, the children with appropriate for gestational age birth weight (AGA) (odds ratio (OR) 1.31; 95% confidence interval (CI) 0.64–2.65) present greater risk for primary hypertension. Infants born with excessive body weight >4000 g irrespective of gestational age, compared to infants born with normal body weight, show increased risk of primary hypertension (OR 1.19; 95% CI 0.68–2.06). Higher risk of hypertension is observed in infants born with greater body length (OR 1.03; 95% CI 0.97–1.08). Conclusions: The problem of hypertension may also affect children with birth weight appropriate for gestational age. The prevalence of hypertension in children with AGA birth weight decreases with age. Birth length can be a potential risk factor for hypertension in children and adolescents.

## 1. Introduction

In recent years, there has been an increase in the number of children and adolescents with primary hypertension (HT). As reported by current literature, the problem relates to 4.7%–19.4% of the young population [1,2]. High values of normal blood pressure (BP), identified in childhood, may persist into adulthood. Development of HT involves a variety of mechanisms resulting from genetic and environmental factors. Regarding this, the role of perinatal factors is increasingly being taken into account [3,4].

A child’s birth parameters not only enable assessment of intrauterine growth but are also very helpful in identifying children at risk of developmental defects or diseases occurring in adulthood.

Epidemiological studies show that children born with body weight that is small for gestational age (SGA) are at a greater risk of primary hypertension [5,6]. In the current literature, the concepts related to the mechanism of high blood pressure occurring in children with low birth weight most frequently take into account endothelial dysfunction, impaired fetal liver function and sympathetic hyperactivity. It is also possible to observe decreased number of nephrons, elevated activity of the renin–angiotensin–aldosterone (RAA) system and impaired function of the hypothalamic–pituitary–adrenal system and, consequently, greater susceptibility to progressing kidney disease [7,8].

The well-established consequences of low body mass at birth occurring in adulthood include a risk—greater than in the overall population—of endocrine and metabolic disorders, such as insulin resistance, abdominal obesity, type II diabetes, as well as coronary heart disease and cerebral hemorrhage [9].

Potentially, the association between birth weight and future susceptibility to obesity may be linked to impaired proportion of fat and lean body mass as well as defective appetite control by the central nervous system [10]. Children born with low body weight are characterized by lower fat body mass in childhood, and the risk of abdominal obesity increases as they grow up [11].

The relation between excessive birth weight and the risk of primary hypertension in children is discussed less frequently. It is known that children born with body weight large for gestational age (LGA) have higher insulin levels and lower adiponectin concentration than children born with body weight appropriate for gestational age (AGA), despite similar body mass index (BMI) values. Adiponectin is a better indicator of insulin resistance in LGA children during adolescence, and it is dependent on birth weight [12]. A systematic literature review showed that the risk of hypertension in individuals with high birth weight (>4000 g) was inversely correlated to age [13]. Other authors observed a greater risk of primary hypertension both in SGA and in LGA children between 15 and 24 years of age but not in children aged 8–14 years [14].

The findings of epidemiological studies seem to suggest that both SGA and LGA subjects presented adverse cardiometabolic profiles in childhood, and the problem seemed to increase during adolescence. These results suggest prolonged duration of insulin resistance and a greater cardiovascular risk from childhood to adolescence [15]. It is supposed that at later age, the effects of birth weight on arterial pressure are less significant compared to the impact of birth length [16,17]. However, the role of these factors is still unclear to a certain degree and, therefore, there is a need for further comprehensive research.

The current study was designed to assess the impact of birth weight and length on primary hypertension occurring in children aged 3–15 years. 

## 2. Materials and Methods 

### 2.1. Participants

The examinations took into account 1000 children attending randomly selected primary schools and kindergartens. The values of neonatal parameters, such as birth weight and length, were retrieved from children’s personal health records (“the red book”). The following eligibility criteria were used for inclusion in the study: consent of the parent/legal guardian and the child for participation in the study, the child’s age between 3 and 15 years, attendance of a school/kindergarten, health status allowing for the examinations to be carried out, and no diagnosis of arterial hypertension.

The following criteria for exclusion from the study group were defined: lack of consent of the parent/legal guardian (n = 70) for the child to take part in the study (n = 35) as well as the child’s age being either below 3 or over 15 years. The subjects were excluded from the group if their red books were not provided (n = 103), or if the information in the red books was incomplete (n = 30). Furthermore, children with diagnosed hypertension or taking high blood pressure medication (n = 15) were also excluded from the study. Ultimately, the analyses took into account n = 747 subjects.

### 2.2. Blood Pressure Assessment

Automated oscillometric measurements of arterial blood pressure were performed using a device from Welch Allyn Inc., 4200B-E2 (Skaneateles Falls, NY, USA) along with a set of cuffs of various widths, intended for both children and adults. The mean ± standard deviation for systolic blood pressure readings was −1.0 ± 4.1 mmHg; at diastolic blood pressure was −0.4 ± 6.1 mmHg. These criteria have been approved by the AAMI (Association for the Advancement of Medical Instrumentation) [18]. Blood pressure was measured three times with an interval of 3 min between measurements and the value was taken as the average of the last two [19].

Classification of arterial blood pressure was performed based on centile grids for blood pressure in children developed in the Polish frames of the OLA and OLAF projects and based on oscillometric measurements [20].

The guidelines published in 2016 by the European Society of Hypertension were followed in the process of classifying arterial blood pressure in the children and adolescents [19].

### 2.3. Assessment of Perinatal Risk Factors

Information related to such perinatal factors as birth weight and length, as well as type of labor and timing of delivery, was retrieved from the child’s personal health record.

The children were divided according to their birth weight, irrespective of gestational age: hypertrophic neonate—body weight over 4000 g; eutrophic neonate—body weight between 2500 and 4000 g; and neonate with low birth weight (LBW)—body weight below 2500 g.

The children were also classified according to their birth weight with respect to gestational age: AGA–body weight appropriate for gestational age; SGA—small for gestational age: below the 5th centile for gestational age; and LGA—large for gestational age: body weight at birth over the 95th centile for gestational age [21]. 

### 2.4. Data Analysis

Data analysis was carried out using selected methods of descriptive statistics and statistical inference. Selected numerical characteristics of the tested parameters were determined: number (n), percentage (%), × (mean), and standard deviation (s).

Kruskal–Wallis tests were used to determine the level of statistical significance (*p* < 0.05). To evaluate the risk of hypertension in children, odds ratios (OR) with 95% confidence interval were used. Calculations were performed with Statistica 10.0 (StatSoft, Inc., Tulsa, OK, USA).

### 2.5. Ethical 

The study design was approved by the local Bioethics Commission, decision no.19/12/2015 dated 2 December 2015.

## 3. Results

The mean birth weight of the children in the study group was 3359 ± 565 g and their mean birth length was 55 ± 3.3 cm. In the study group, greater birth weight and length was identified in the male infants, compared to the female infants (3430 g vs. 3281 g, *p* < 0.001; 55 cm vs. 54.8 cm, *p* = 0.049).

Nearly 92% of the children were born with appropriate weight for gestational age (AGA), 7.4% of the children were born small for gestational age (SGA) and nearly 1% of the children were born large for gestational age (LGA). The largest part of the group (84.5%) comprised eutrophic neonates, with body weight ranging from 2500 to 4000 g. The smallest part of the group (5.5%) comprised children with low birth weight, below 2500 g. The infants with high birth weight accounted for 10.1% of the group (Table 1).

The birth weight of the children in the group with hypertension is slightly higher than in the case of the children with normal blood pressure, though the relationship is statistically insignificant. The findings show no statistically significant (but close to statistical significance) association between blood pressure and the child’s birth length (*p* = 0.066) (Table 2).

A Kruskal–Wallis ANOVA test was used to analyze the differences in body length of newborns from three groups. A nonparametric test was used due to the lack of normality of distributions for the analyzed variable in three groups. The obtained result *p* = 0.066 at the adopted statistical significance threshold *p* < 0.05 indicated no differences between the results in the groups. Similar medians (55 vs. 55 vs. 56) confirmed the absence of statistically significant differences between the groups.

The presence of a statistically significant linear relationship between the body length of newborns and the value of their arterial pressure, both systolic and diastolic (*p* = 0.064 and *p* = 0.081), was not confirmed. The relationship between variables was analyzed using the Spearman’s rank correlation test (Table 3).

In the group of children aged 4–6 years, a difference approaching statistical significance was found in the prevalence of hypertension identified in AGA children (29.1%) compared to SGA children (5.9%) (*p* = 0.088). It is also possible to notice a decreasing frequency of hypertension in AGA children with age. In the youngest age group, the relevant rate amounted to 29.1%, and it decreased to 15.1% in the group of subjects aged 12–15 years. Likewise, in the SGA group hypertension was found more frequently in children aged 7–11 years compared to the older subjects (Table 4).

The analyses showed that the children born with high body weight, irrespective of the gestational age, were more frequently identified with hypertension (25.3%), although the difference was not statistically significant (*p* = 0.666) (Table 5).

Higher risk of hypertension was observed in infants born with greater body length (OR 1.03; 95% CI 0.97–1.08) (Table 6).

Compared to the SGA children, the children in the AGA group (OR 1.31; 95% CI 0.64–2.65) present greater risk for primary hypertension (Table 6). The children with low birth weight (LBW) (OR 0.6; 95% CI 0.25–1.46) show decreased risk of primary hypertension compared to the children with normal birth weight 2500–4000 g (eutrophic neonates). Infants born with high body weight, >4000 g, compared to infants born with normal body weight show increased risk of primary hypertension (OR 1.19; 95% CI 0.68–2.06) (Table 6). Due to its small size, the LGA group was disregarded.

## 4. Discussion

Many authors have made attempts to describe the association between birth parameters and the prevalence of hypertension in children. Studies have shown that the problem of hypertension may be present in children who were born with normal body weight and length. While most of the available studies primarily focus on the relationship of low or high birth weight, as well as the onset of hypertension during adulthood, our own research indicates an equally great need to study children who had appropriate weight for gestational age. The results suggest that the etiology of primary hypertension is multifactorial and that perinatal parameters are not the only ones that are involved in the genesis of this problem. It should be added that the number of LGA and SGA groups in the present study was much lower than in the AGA group and, hence, it cannot be concluded that the risk was significantly higher in children with AGA than LGA and SGA. Comparison of these groups was not the primary goal of the presented study and requires further confirmation through research on a larger group of subjects with LGA and SGA.

A relationship approaching statistical significance was also found between hypertension and the child’s birth length. The children with hypertension were found to have slightly greater birth length, compared to the children with normal blood pressure. Similarly, Krzych et al. in their study did not identify a relationship between hypertension and birth weight, but they reported a mild, though statistically significant effect of birth length in the relevant group of children aged 7–18 years. Each increase in the length by one centimeter was linked with an increase in systolic pressure by 0.05 mmHg, and in diastolic pressure by 0.07 mmHg. The lack of a relationship between blood pressure and birth weight has also been reported by Steinthorsdottir et al. [3].

The present findings are in opposition to most research reports which show an inverse association between the value of arterial pressure and birth weight. 

A meta-analysis of 20 clinical studies showed a 21% higher risk of hypertension occurring in adulthood in subjects with low birth weight compared to individuals with normal weight at birth. The subjects with birth weight below 2500 g were characterized by systolic pressure higher by 2.6 mmHg [22]. Shankaran et al. found, in a study involving 6-year-old children, that hypertension was more likely to occur in children with confirmed intrauterine growth restriction compared to a control group [23]. A study by Yiu reported that each decrease in birth weight by 1 kg corresponded in infants to a 1.3 mmHg increase in systolic pressure and 0.6 mmHg increase in diastolic pressure [24]. Likewise, Polish authors also showed that at the age of 6–10 years, children with low birth weight were more likely to have hypertension or high normal blood pressure, compared to AGA children (50% vs. 16%, *p* < 0.01) [25].

Furthermore, Pocobelli et al. reported that compared to AGA children, the risk of hypertension is greater in SGA subjects (OR 1.32; 95% CI 1.02–1.71) and in LGA subjects (OR 1.30; 95% CI 1.00–1.71). These results also suggest a nonlinear relationship between birth weight and the risk of primary hypertension in children and young adults. These effects were found in examinations conducted at 15–24 years of age, but such an association was not identified at 8–14 years of age [14]. Moreover, a study by Kuhle et al. showed that at the age of 6–12 years, the values of anthropometric parameters were lower in the SGA children and higher in the LGA children in comparison to the AGA group, yet most of the differences were not statistically significant. The findings, however, did not show differences related to arterial pressure between SGA or LGA infants and the AGA group [26].

In the present study, hypertension was most frequently observed in the AGA children (22.5%). In the SGA group, hypertension was found in 18.2%, and in the LGA group in 14.3% of the subjects. In the group of children aged 4–6 years, the difference was approaching statistical significance; hypertension was identified in 29.1% of the children born with appropriate weight for gestational age AGA, compared to the children born small for gestational age SGA—5.9% (*p* = 0.0875). Additionally, it was observed that the likelihood of hypertension in the AGA group decreased with age. In the youngest group and the oldest group, the relevant rates were 29.1% and 15.1% respectively. 

Similar findings were reported by Zhang et al. The mean difference in blood pressure and the relative risk of hypertension in subjects born with normal body weight and with high body weight were inversely associated with age. Systolic and diastolic pressure, as well as the likelihood of hypertension, were higher in younger children with high birth weight but lower than in older subjects with the same type of birth weight. The findings suggest that individuals with high birth weight are more susceptible to hypertension and high blood pressure in childhood. However, in the case of these subjects, the elevated blood pressure tends to “drop” as they get older. Hence, older individuals with high birth weight are less prone to hypertension than individuals with normal birth weight [13].

Most researchers seem to agree that low birth weight predisposes for higher blood pressure levels in childhood. A study involving Lithuanian adolescents also showed a relationship between high blood pressure and being born large for gestational age (LGA). The largest likelihood of high blood pressure was identified in individuals born with excessive body weight and those with overweight or obesity during adolescence [27]. 

Due to the small number of children in the SGA and LGA groups in the surveyed population, the results of the AGA group and their analysis are the most reliable. The presented results from the SGA and LGA groups show some trends in the occurrence of hypertension in these children, though they require further studies in a larger study group. However, this does not change the fact that hypertension in the AGA group was observed in as much as 22.5% of respondents and it was the highest result. In view of the conflicting findings, further research in this area is needed. Furthermore, given the fact that hypertension occurring in childhood tends to decrease in adulthood, it is necessary to investigate this type of relationships in adulthood.

## 5. Conclusions

- The problem of hypertension may also affect children with birth weight appropriate for gestational age.
- The prevalence of hypertension in children with birth weight appropriate for gestational age decreases with age.
- Birth length can be a potential risk factor for hypertension in children and adolescents.

## Figures and Tables

**Table 1:** Characteristics of perinatal parameters in the study group. LBW, low birth weight; AGA, appropriate weight for gestational age; SGA, small for gestational age; LGA, large for gestational age.

Children’s Characteristics	Value
Sex ^a^	
Male	390
Female	357
Birth weight for gestational age ^a^	
AGA	685
SGA	55
LGA	7
Birth weight ^a^	
LBW < 2500 g	41
Eutrophic neonate 2500–4000 g	630
Hypertrophic neonate > 4000 g	76
Timing of delivery ^a^	
<37th week of gestation	122
>37th week of gestation	625
Type of delivery ^a^	
natural birth	438
caesarean section	309
Systolic blood pressure ^b^	
AGA	110.9 (±11.53)
SGA	107.3 (±13.70)
LGA	108.43 (±10.04)
Diastolic blood pressure ^b^	
AGA	68.7 (±9.74)
SGA	66.9 (±8.66)
LGA	63.6 (±5.53)

^a^ number of participants; ^b^ average ± standard deviation.

**Table 2:** Child’s birth parameters versus arterial blood pressure.

Child’s Birth Parameters	Blood Pressure Classification												*p*
	Normal Blood Pressure				High Normal Blood Pressure				Hypertension				
	*N*	$\bar{x}$	Me	*s*	*N*	$\bar{x}$	Me	*s*	*N*	$\bar{x}$	Me	*s*	
Child’s birth weight (g)	444	3358	3400	557	138	3314	3400	580	165	3397	3480	577	0.258
Child’s birth length (cm)	442	55.1	55	3.2	138	54.6	55	3.1	164	55.2	56	3.5	0.066

**Table 3:** Correlation analysis between blood pressure values and body length of newborns.

	R	*p*
Birth length vs. systolic blood pressure	0.07	0.064
Birth length vs. diastolic blood pressure	0.06	0.081

R—Spearman’s rank correlation test result; *p*—significance level.

**Table 4:** Classification of birth weight versus hypertension in the age groups.

	Age Group 4–6 Years (*p* = 0.088)		
Blood Pressure Classification	Centile-Based Classification of Birth Weight		
	SGA	AGA	LGA
Normal blood pressure	12 (70.6%)	73 (46.2%)	3 (100.0%)
High normal blood pressure	4 (23.5%)	39 (24.7%)	0 (0.0%)
Hypertension	1 (5.9%)	46 (29.1%)	0 (0.0%)
Total	17	158	3
	Age Group 7–11 Years (*p* = 0.836)		
Normal blood pressure	14 (51.9%)	192 (58.5%)	3 (75.0%)
High normal blood pressure	6 (22.2%)	58 (17.7%)	0 (0.0%)
Hypertension	7 (25.9%)	78 (23.8%)	1 (25.0%)
Total	27	328	4
	Age Group 12–15 Years (*p* = 0.894)		
Normal blood pressure	7 (63.6%)	140 (70.4%)	0
High normal blood pressure	2 (18.2%)	29 (14.6%)	0
Hypertension	2 (18.2%)	30 (15.1%)	0
Total	11	199	0

**Table 5:** Birth weight category versus hypertension in the children.

Blood Pressure Classification	Birth Weight Classification (*p* = 0.666)		
	Hypertrophic	Eutrophic	LBW
Normal blood pressure	45 (60.0%)	371 (58.9%)	27 (65.9%)
High normal blood pressure	11 (14.7%)	119 (18.9%)	8 (19.5%)
Hypertension	19 (25.3%)	140 (22.2%)	6 (14.6%)
Total	75	630	41

**Table 6:** Risk of hypertension in children, relative to birth length, birth weight as well as birth weight for gestational age.

	Number of Children n = 747			
	N	%	OR	95% CI
Birth length	164	22	1.03	0.97–1.08
Birth weight				
LBW < 2500 g	6	14.6	0.60	0.25–1.46
Eutrophic neonate 2500–4000 g	140	22.2	1.00	Reference group
Hypertrophic neonate >4000 g	19	25.3	1.19	0.68–2.06
Birth weight for gestational age				
SGA	10	18.2	1.00	Reference group
AGA	154	22.5	1.31	0.64–2.65
LGA	1	14.3	−	−

*p*—probability value calculated using chi-square independence test; *OR*—odds ratio (with 95% confidence interval).
